# Searching for metastable particles using graph computing

**DOI:** 10.1038/s41598-021-97848-6

**Published:** 2021-09-17

**Authors:** Ashutosh V. Kotwal

**Affiliations:** Department of Physics, Duke University, Durham, NC 27708 USA

**Keywords:** Computational science, Experimental particle physics

## Abstract

The reconstruction of charged particle trajectories at the Large Hadron Collider and future colliders relies on energy depositions in sensors placed at distances ranging from a centimeter to a meter from the colliding beams. We propose a method of detecting charged particles that decay invisibly after traversing a short distance of about 25 cm inside the experimental apparatus. One of the decay products may constitute the dark matter known to be 84% of all matter at galactic and cosmological distance scales. Our method uses graph computing to cluster spacepoints recorded by two-dimensional silicon pixel sensors into mathematically-defined patterns. The algorithm may be implemented on silicon-based integrated circuits using field-programmable gate array technology to augment or replace traditional computing platforms.

## Introduction

The discovery of the Higgs boson^1–4^ at the Large Hadron Collider (LHC)^5,6^ has confirmed one of the most important building blocks in the relativistic quantum field theory of fundamental particles and their interactions—the standard model (SM) of particle physics^7–9^. However, despite its enormous success in describing and predicting a vast number of phenomena, the SM is far from being a complete theory. The gravitational interaction of dark matter (DM) on the galactic and cosmological distance scales^10–12^ is crucial for large-scale structure formation. Cosmological data are consistent with DM comprising about 84% of the matter in the universe^13^. Dwarf galaxies comprised mostly of DM have recently been discovered^14^. However, DM cannot be accounted for in the SM, which reveals one of the sources of incompleteness of the SM.

It is plausible that DM is comprised of one or more new species of particles^15^. The LHC may copiously produce metastable charged partners of the DM particles^16,17^ if they form a nearly-degenerate symmetry group multiplet under the electroweak interaction^18–22^. In this case the decay of the charged progenitor to the invisible DM will produce negligible associated energy, which makes the identification of these metastable charged particles in a short time interval commensurate with the collision rate a challenging task. Research and development is ongoing in this area^23–40^. Triggering on charged particles is currently based on their energy deposition in the calorimeters surrounding the tracking sensors, or on their ability to penetrate the calorimeters and shielding and reach the outermost sensors. The former case includes high-momentum electrons and positrons which have a sufficiently low mass to create in the calorimeter an electromagnetic cascade of radiated photons and their subsequent conversion to electron–positron pairs. It also includes pions, kaons and protons whose strong interactions with atomic nuclei create a hadronic cascade in the calorimeter. The latter case includes muons which are too massive to create an electromagnetic cascade and do not interact strongly with nuclei to produce a hadronic cascade. Thus, muons deposit a small amount of ionization energy in the calorimeters and penetrate the shielding to trigger the outermost muon sensors. In some models of new physics, a long-lived particle may induce a hadronic cascade that is not fully contained in the calorimeter, and the particles that leak out of the back of the calorimeter can trigger the muon sensors.

**Figure 1:**
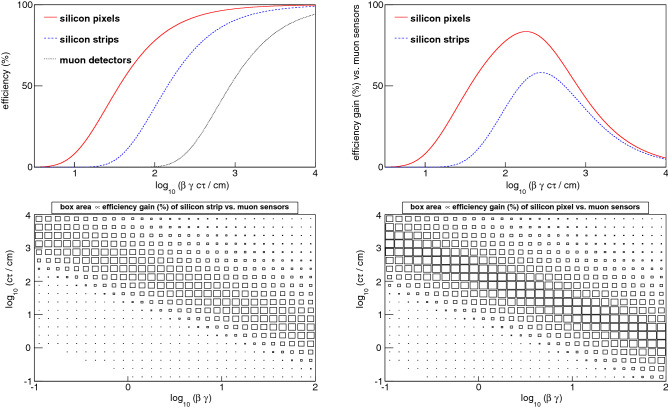
(top left) The probability of a metastable particle to traverse the silicon pixel sensors ($r_{\max } = 25$ cm), silicon strip sensors ($r_{\max } = 1$ m), and the muon sensors ($r_{{\rm typical}} = 6$ m), before decaying. (top right) The incremental gain in triggering efficiency by deploying our method on the silicon tracking sensors, given existing muon sensor triggers. The incremental gain is defined as the difference between the pixel or strip sensor curve and the muon sensor curve in the top-left figure. Note that the muon sensors are located at radii between 4.5 m and 7 m (10 m) in CMS (ATLAS). (bottom) The incremental gain in triggering efficiency by using the silicon strip (left) and pixel (right) sensors, in the phase space of $\beta \gamma ~ (= p/m)$ and $c \tau$. The probability in each bin is proportional to the box area, on a scale of 0–100%.

In this paper we describe a triggering scheme for “disappearing tracks”, which are sufficiently massive to interact like muons but not sufficiently long-lived to reach the muon sensors. The length of the charged-particle trajectory before its decay is distributed exponentially with a mean value of $\beta \gamma c \tau$, where $\beta \equiv v/c$, *v*(*c*) is the particle (light) speed, $\gamma$ is the Lorentz time-dilation factor $\gamma \equiv (1 - \beta ^2)^{-\frac{1}{2}}$, and $\tau$ is the particle’s proper lifetime. The probability for a metastable particle to decay beyond 25 cm, 1 m and 6 m of flight distance is shown in Fig. 1. These distances correspond, respectively, to the outer radius of silicon pixel sensors, the outer radius of silicon strip sensors, and the typical radius of the muon sensors, in the ATLAS^5^ and CMS^6^ experiments at the LHC. We note the substantial increase in the parameter phase space over which triggering is enabled by utilizing solely the silicon tracking sensors.

## Physics motivation

As an example, a theoretical model that motivates our method is based on supersymmetric partners of the photon, $W^{\pm }, Z$ and Higgs bosons, the former being the mediators of the electroweak interaction. As discussed in Ref.^19–21^, the supersymmetric partners (“winos”) of the $W^{\pm }$ bosons may be almost degenerate in mass with the lightest supersymmetric particle (LSP). In this scenario the metastable wino ($c \tau \sim 6$ cm independent of mass) decays to a charged pion of very low energy and the stable, neutral LSP which is a dark-matter candidate. Figure 1 shows that, for the same triggering probability, the momentum *p* of a wino of mass *m* can be a factor of $\approx 25$ smaller if the wino is triggered using the silicon pixel sensors rather than the muon sensors. A wino with $m = 100$ GeV, $p = 500$ GeV and $c \tau \sim 6$ cm would have a trigger efficiency of 50% with the pixel sensor-based track trigger that we demonstrate in this paper. To achieve the same efficiency, $p>12$ TeV would be required by the muon trigger, for which the production rate is vanishing. This example illustrates the benefit of using our method to devise a trigger on charged particles using only the tracking sensors at small radius, since the production rate at the smaller momentum will be orders of magnitude larger.

**Figure 2:**
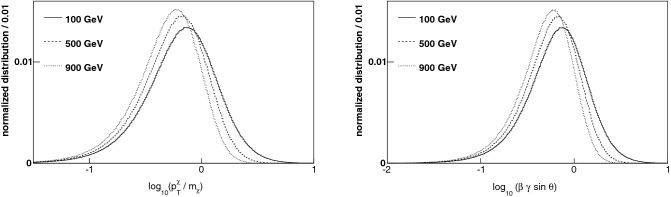
(Left) The distribution of transverse momentum $p_T$ of charged particles $\chi ^\pm$ pair-produced at the LHC via the Drell-Yan process, calculated using the pythia 8.219 event generator^50^ and the nnpdf3.1 set of parton distribution functions^51^. The approximate scaling property of this distribution is shown by plotting $p_T / m_\chi$, where $m_\chi$ is the particle mass. (right) The corresponding distributions of the quantity $\beta \gamma \sin \theta$, which takes into account both the Lorentz-boost and the path-length factors relevant for the trigger efficiency with cylindrical detectors. The distributions vary little over the wide range $100< m_\chi < 900$ GeV. The typical boost for lighter particles is slightly larger that the boost for heavier particles.

We quantify the gain in sensitivity by integrating the trigger efficiencies over the momentum spectrum of massive charged particles produced at the LHC. Pair production of weakly-interacting charged particles, denoted generically by $\chi ^\pm$, is initiated by quark–antiquark annihilation and mediated by *s*-channel virtual-photon and *Z*-boson exchange in the Drell-Yan process $q \bar{q} \rightarrow \gamma ^*/Z^* \rightarrow \chi ^+ \chi ^-$. The production rate depends only on the mass of the $\chi$ particle and its weak charge and/or hypercharge which determines its coupling to the *Z*-boson; the kinematics are fairly model-independent and shown in Fig. 2. In particular, the product of the time-dilation factor $\gamma$ and the transverse velocity $\beta \sin \theta$, which determines the relevant path-length before decay, has a distribution which is approximately independent of the particle mass $m_\chi$. Small values of $\beta$ are phase-space suppressed while large values of $\gamma$ are suppressed by the virtuality of the mediator and the softness of the quark and antiquark distributions.

**Figure 3:**
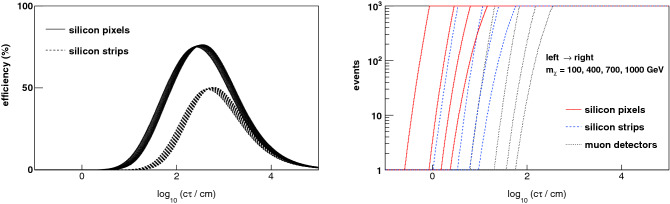
(Left) The effective trigger efficiencies (averaged over the boost spectrum) as functions of the proper decay distance $c \tau$ for a range of masses $100< m_\chi < 1000$ GeV, showing the small dependence on $m_\chi$. The difference between the spectrum-convolved efficiency of a pixel- or strip-based trigger, and the same for a muon-based trigger, is shown. The efficiency gain curves are shifted to lower values of $c \tau$ for lower values of $m_\chi$. (right) Based on the effective trigger efficiencies, the number of events observable for an integrated luminosity of 3 ab$^{-1}$ at the HL-LHC is shown as a function of $c \tau$ for pair production of supersymmetric partners of leptons. For each trigger system, the four curves (from left to right) correspond to supersymmetric lepton masses of 100, 400, 700 and 1000 GeV respectively.

**Figure 4:**
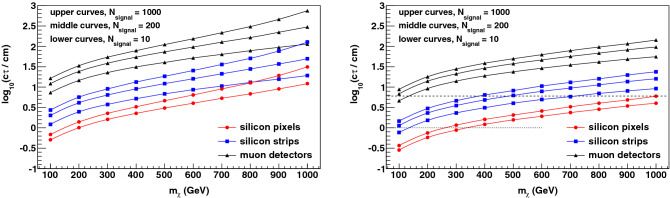
(Left) The discovery reach in $c \tau$ for metastable supersymmetric leptons of mass $m_\chi$ for an integrated luminosity of 3 ab$^{-1}$ at the HL-LHC. The values of $c \tau$ above the curves are in the discoverable range, defined as the observation of 200 (1000) signal events for the middle (upper) curve with a given trigger system. For the muon- and silicon strip-based triggers, an additional (lower) curve corresponding to 10 signal events is also shown. (right) The corresponding discovery reach for metastable winos and higgsinos. The theoretically motivated value of $c \tau \sim 6$ cm for the nearly-degenerate wino’s proper decay distance^19–21^ is indicated by the dashed horizontal line. It is clear that this illustrative model is rendered discoverable for wino mass values beyond 1 TeV by a pixel-based track trigger. The theoretically motivated value of $c \tau \sim 1$ cm for the nearly-degenerate higgsino’s proper decay distance^20,22^ is indicated by the dotted horizontal line. The short-lived higgsino’s discovery in a motivated range of masses is enabled by the track trigger.

Integrating the trigger efficiencies over the respective spectrum of the boost and path-length factor for each $m_\chi$ yields the effective trigger efficiencies as functions of the proper decay distance $c \tau$ of the $\chi$ particle. Given the approximate invariance of the boost spectrum, the effective trigger efficiencies are fairly independent of $m_\chi$, as shown in Fig. 3. Since the typical value of $\beta \gamma$ is $\mathcal {O}$(1), the differential efficiency gain with respect to a muon trigger is visible in the range 10 cm $< c \tau<$ 10 m. From the perspective of event rates from new physics, it is also interesting to consider the ratio of the silicon-trigger efficiency to the muon-trigger efficiency. This ratio is large for $c \tau \sim 1$ cm even though all efficiencies are small. Thus, depending on the $\chi$-production cross section and the integrated luminosity, the triggered event rate at these small values of $c \tau$ may be raised above the discovery threshold by a silicon-based track trigger. To illustrate, we consider the pair-production of supersymmetric partners of leptons (“sleptons”) and winos for $\sqrt{s} = 13$ TeV at the LHC. In the mass range $100< m_\chi < 1000$ GeV, the cross section ranges from 366 fb to 15 ab (11.6 pb to 622 ab) for slepton (wino) pair production^41–49^. Based on the effective trigger efficiencies, the number of slepton events observable for an integrated luminosity of 3 ab$^{-1}$ at the high-luminosity LHC (HL-LHC) is shown as a function of $c \tau$ in Fig. 3. A silicon pixel-based trigger enables a substantial increase in event rate down to low values of $c \tau$. The discovery reach in $c \tau$ for a range of slepton and wino masses is summarized in Fig. 4, for a discovery threshold defined as the observation of 200 or 1000 signal events. The theoretically motivated value of $c \tau \sim 6$ cm for the nearly-degenerate wino’s proper decay distance^19–21^ is rendered discoverable for wino mass values beyond 1 TeV by a pixel-based track trigger. Another model prediction is nearly-degenerate “higgsinos”, supersymmetric partners of Higgs bosons, with a shorter proper lifetime of 7–14 mm^20,22^ for the charged higgsino and the lightest higgsino being the neutral and stable DM particle. Fig. 4 illustrates the physics reach for the charged higgsino with the pixel-based track trigger.

**Figure 5:**
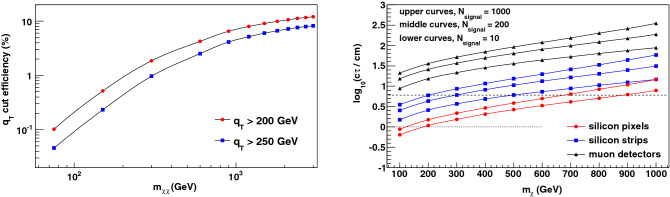
(Left) The efficiency of requiring a minimum $\chi \chi$-system transverse momentum $q_T$, imparted by initial-state QCD radiation (ISR). The efficiency is estimated using the dyqt program^61,62^ for $\sqrt{s} = 13$ TeV at the LHC and is shown as a function of the system invariant mass. (right) The reduced discovery reach for metastable winos and higgsinos, after folding in the efficiency of the minimum $q_T$ requirement, to be compared to Fig. 4, also for an integrated luminosity of 3 ab$^{-1}$ at the high-luminosity LHC.

In the absence of a track trigger, the initial-state QCD radiation (ISR) accompanying $\chi$-pair production can provide a trigger, as demonstrated in^52–54^. The disadvantage of this strategy is a substantial loss of acceptance, since the total transverse momentum $q_T$ of the ISR is required to be large^52–54^ and the $q_T$ spectrum is soft. We use the dyqt program^61,62^ to estimate the efficiency of the minimum-$q_T$ requirement. The dyqt program performs the perturbative QCD calculation of Drell-Yan production up to $\mathcal O(\alpha _s^2)$ at high values of $q_T$, where $\alpha _s$ is the QCD coupling, and resums the logarithmically-enhanced $\mathcal O[\alpha _s^n \ln ^m(q_T^2/m_{\chi \chi }^2)]$ QCD contributions at small values of $q_T$ up to next-to-next-leading logarithmic terms. The resulting efficiency is shown in Fig. 5, and ranges from 0.1% or less for $m_{\chi \chi } < 100$ GeV to about 10% at high $\chi$-pair invariant mass. Since the observation of metastable charged particles requires their passage through the tracking detectors, the minimum-$q_T$ requirement’s efficiency factors directly into the observable rate. The resulting reduced rate and the loss of discovery potential is shown in Fig. 5, providing strong motivation for a pixel-based track trigger. Compared to the ISR trigger, the track trigger increases the observable signal rate by a factor of 10–1000, which, at the minimum, is equivalent to the increase in integrated luminosity provided by the entire HL-LHC run. A comparison of Figs. 4 and 5 shows that a track trigger can achieve a given signal yield at a factor of 2–3 lower lifetime over a broad range of masses, and can increase the mass reach for a motivated range of lifetimes. The example of the nearly-degenerate winos illustrates the gain at high mass. The increase in sensitivity at low masses is also visible when considering the example of the shorter-lived higgsino.

The chosen discovery thresholds for signal yield are illustrative, motivated by the disappearing-track analysis published by the CMS Collaboration^54^. Using 101 fb$^{-1}$ of integrated luminosity and the ISR trigger strategy, the analysis found the data to be consistent with the estimated background of $48 \pm 9$ events. The backgrounds are comprised of misreconstructed high-$p_T$ leptons from electroweak processes and spurious tracks from largely random hit combinations. It is shown that these background processes are insensitive to event topology features such as the ISR trigger activity. Since the LHC experiments are being upgraded^55–58^ for the HL-LHC to maintain, and likely improve upon, the current detector performance, a conservative estimate of the background, scaled to an integrated luminosity of 3 ab$^{-1}$ is 1,500 events with the ISR-trigger strategy, requiring the observation of 200 signal events for a discovery of $5 \sigma$ statistical significance.

As a track trigger has never been deployed at the ATLAS or CMS experiments, background estimates without the ISR-trigger requirements have not been published since such an analysis has not been possible. In the absence of this information, we perform an estimation based on the dyqt calculation mentioned above. Since the CMS analysis^54^ required at least one jet with $p_T > 110$ GeV, we use dyqt to estimate the fraction of electroweak-boson production events satisfying this requirement. This fraction of 4% implies that a track trigger would experience a $25 \times$ larger prompt-lepton background than the ISR trigger. Furthermore, it is shown in the CMS analysis^54^ that the spurious-track background is approximately equal to the prompt-lepton background for tracks with five hits, which is guaranteed by our proposed method. Thus, the total background estimate for the track trigger is 38,000 events, requiring the observation of 1,000 signal events for a discovery of $5 \sigma$ statistical significance. The availability of such high statistics will enable improvements in analysis and background-reduction techniques, and better control of systematic uncertainties, that will no doubt be needed to exploit the gain in statistical power. Based on these estimates, we have chosen signal event yields of 200 and 1000 events as illustrative discovery thresholds.

We emphasize that an analysis based solely on event counting, on which the above estimates are based, is likely to grossly underestimate the ultimate sensitivity that can be achieved with the track trigger. By providing 10–100$\times$ more acceptance than the ISR trigger, the track trigger will enable a differential analysis, exploiting the difference between signal and background distributions. For example, the track $p_T$ distribution for the prompt-lepton background is expected to fall rapidly with increasing $p_T$, while the signal yield peaks near $p_T \sim m_\chi$. Spurious-track backgrounds are expected to increase with pseudorapidity, while the signal yield peaks at low pseudorapidity. Thus, the search for a high-mass signal can be reoptimized with much lower background than the estimates presented above based on an inclusive analysis. With lower backgrounds, the large acceptance increase will yield correspondingly larger physics sensitivity gains. As a simple rule of thumb, an acceptance increase of a factor of *g* increases the signal significance by a factor of $\sqrt{g}$ for a background-dominated analysis, but increases the significance by a factor of *g* when the background can be made negligible while maintaining an observable signal yield. Such optimization is enabled by a large acceptance trigger such as the proposed track trigger.

An estimate of the background for the muon-based trigger is provided by the ATLAS Collaboration’s search for heavy charged long-lived particles using 36 fb$^{-1}$ of integrated luminosity^59^. Searching for pair-produced winos of mass above 200 GeV, a background of 230 events was estimated, after suppressing prompt muons using time-of-flight information from the muon detectors. Simply scaling this background estimate to 3 ab$^{-1}$ implies that a $5 \sigma$ statistical significance would require the observation of 700 signal events. Allowing for a larger or smaller background with the upgraded detectors and higher pileup conditions, our illustrative discovery thresholds of 200 and 1000 signal events are also appropriate for a muon-triggered search. Again, we emphasize that backgrounds can be strongly suppressed by applying high-$p_T$ requirements, as demonstrated in this ATLAS analysis^59^, where the estimated background reduces by a factor of 2–3 for a 100 GeV increase in the wino mass. This justifies our statement that at sufficiently high mass, the search is not background-limited but is rate-limited, and benefits more strongly from the substantially higher acceptance of the proposed track trigger. A preliminary ATLAS analysis released recently^60^ also demonstrates that disappearing-track backgrounds are expected to reduce by one-two orders of magnitude as the track $p_T$ increases from 100 to 1000 GeV.

In any case, the substantial extension of the physics reach to lower lifetimes is clearly visible in Fig. 4, regardless of the background level in the muon trigger. Even under the aggressive assumption that backgrounds could be very strongly suppressed to $\mathcal O$(1 event) for the muon trigger in the future, and a discovery could be made with as few as 10 signal events observed, Fig. 4 shows that the pixel trigger always has substantially higher sensitivity than the muon trigger, in the proper decay-distance range of a few mm to a few tens of cm for all wino and slepton masses considered.

The same conclusion can be drawn for the silicon strip-based trigger. Since such a trigger has never been deployed, an analysis studying the expected backgrounds for this trigger is not published. The background from spurious tracks has been shown to decrease, while the background from leptons has been shown to increase, for the longer strip tracks compared to the shorter pixel tracks in the CMS analysis^54^. Nevertheless, Fig. 4 also shows the discovery potential for a strip-based trigger for an aggressive assumption of a very low background of $\mathcal O$(1 event) and a signal yield of 10 events. Even in this unrealistic scenario, the pixel-based trigger extends the physics reach to a $2 \times$ lower lifetime compared to the strip-based trigger.

A final word on the muon trigger - as the particle has already reached the muon detectors and no subsequent measurements along the trajectory exist, a lifetime measurement is not possible since the reduction in rate with increasing decay distance cannot be measured. In comparison, the pixel-based track trigger provides data on the number of outer silicon-strip sensors traversed, and the subset of candidates reaching the muon detectors, before decaying. Thus, the pixel-based trigger enables the measurement of lifetime, which is of great importance.

In summary, a silicon pixel-based track trigger clearly offers an extended physics reach for promptly-produced metastable particles, in comparison to silicon strip-based and muon-based triggers. In comparison to ISR triggers, the pixel-based trigger has substantially larger signal acceptance and a larger background as a consequence. Comparing Figs. 4 and 5, the short-lived higgsino sensitivity increases from 200 GeV with the ISR trigger to 300 GeV with the pixel trigger, taking into account the increased background rate. The longer-lived wino sensitivity increases from 900 GeV to 1 TeV, similarly taking into account the background rate increase. In both cases, taking the rapidly-falling $p_T$ spectrum of the background and the signal-to-background differences of pseudorapidity distributions into account will further increase the realizable gains, as long as there is sufficient signal yield.

## Method

Our method can use data from the two-dimensional pixel sensors and from the one-dimensional strip sensors, both of which are arranged in concentric cylinders surrounding the colliding beams at the center of the LHC detectors^5,6^. In this paper we present results based on the pixel sensors since their acceptance extends over a larger range of the metastable particle’s momentum and lifetime. Each cylindrical surface is covered by arrays of pixels with each dimension of $\mathcal O$(100) $\upmu$m. Electric charge deposited by particles in the traversed pixels is recorded to provide three-dimensional spacepoints along their helical trajectories in the axial magnetic field^5,6^. The HL-LHC will produce about 200 proton-proton collisions every 25 ns^63^, each collision producing about 70 charged particles^64^.

Helical trajectories are described by the azimuthal $\phi (r)$ and longitudinal *z*(*r*) coordinates, where *r* represents the cylindrical radius from the beam axis. Momentum perpendicular to the beam (*z*) direction, $p_T \propto B R$ where *B* denotes the strength of the magnetic field and *R* is the helix radius. These helix coordinates can be calculated as

1 $$\begin{aligned} \sin (\phi - \phi _0)= & {} cr \nonumber \\ z - z_0 = \frac{\lambda }{c} (\phi - \phi _0)= & {} \frac{\lambda }{c} \sin ^{-1} (cr) \end{aligned}$$

where the curvature $c \equiv q/(2R)$, *q* is the particle charge, and the constants $\phi _0$, $\lambda$ and $z_0$ specify the azimuthal angle, the cotangent of the polar angle and the *z*-position of the particle at emission, respectively. Each spacepoint measurement is denoted by *h*(*r*) with attributes of $\phi (r)$ and *z*(*r*) respectively.

## Algorithm

The collection of spacepoints *h*(*r*) created by all charged particles is represented^65^ by a matrix of spacepoints $h_{i,l}$ where *l* denotes the sensor layer ($l \in \{0,1,2\ldots M-1\}$ increasing with radius for *M* layers) and *i* denotes the point’s ordinal number in that layer. Each spacepoint $h_{i,l}$ is associated with $(\phi ,z)$ measurements in layer *l* as mentioned above, and is referred to as a “hit”.

Our algorithm makes use of the methods of graph computing, wherein each data point is considered a vertex in a graph and is linked to adjacent vertices. Early work on graph theory was done by Leonhard Euler. An algorithm to find the shortest distance between nodes on a graph with positive link weights was invented by Edsger W. Dijkstra in 1956^66^. We use the definitions of derivatives on a graph that are provided in Ref.^67^, though we are not training graph neural networks per Ref.^67^ nor are we using supervised machine learning of any kind. The $h_{i,l}$ matrix is converted into a graph by associating link weights $w_{ij,l}$ between each hit $h_{i,l}$ and all possible hits $h_{j,l+1}$ in the next outer layer, where $w_{ij,l} \propto (r_{l+1} - r_{l})^{-1}$^67^. Reconstruction of particle trajectories is achieved by eliminating all spurious links in the $w_{ij,l}$ matrix, such that the surviving links connect the hits associated with a physical particle trajectory.

This graph computing problem is solved by defining the graph operator $\Box _{ijk,l}$ at each node (*i*, *l*) using the triplet of hits $h_{i,l}$, $h_{j,l+1}$ and $h_{k,l-1}$. From Eq. 1 it is straightforward to show^65^ that for the high $p_T$ ($c \rightarrow 0$) particles of interest, $d \phi / dr = \phi ' \rightarrow c$ and $d^2 \phi / dr^2 = \phi '' \rightarrow rc^3$, hence $[\phi '' - r (\phi ')^3] \rightarrow 0$. Similarly, Eq. 1 yields^65^ $d z / dr = z' \rightarrow \lambda$ and $d^2 z / dr^2 = z'' \rightarrow rc^2\lambda$ for high-$p_T$ particles, and $[z'' - r (\phi ')^2 z'] \rightarrow 0$. The criterion for finding valid trajectories is therefore the simultaneous minimization of $| \phi '' - r (\phi ')^3 |$ and $| z'' - r (\phi ')^2 z' |$ at each point of the graph, which is equivalent to the minimization of the exact operator $( | \phi '' - r (\phi ')^3 | + | z'' - r (\phi ')^2 z' | )$^65^.

In the high-$p_T$ limit the computation of the exact operator can be simplified to the linear graph operator $\Box _{ijk,l} = | \phi ''_{ijk,l} | + | z''_{ijk,l} |$ (for $l \in \{1,2\ldots M-2\}$). Our results show that, in comparison to the exact operator, this simplified $\Box _{ijk,l}$ operator gives excellent results when applied to the HL-LHC pixel-detector configuration^55,56^ of five sensor layers with a radial spacing of 5 cm, while improving the computational efficiency by neglecting the non-linear $(\phi ')^3$ and $(\phi ')^2 z'$ terms. The first derivatives are computed as the link-weighted differences of $\phi$ or *z* values at two hits connected by a link, and the second derivatives are computed at a middle layer as the respective differences of first derivatives to the next layer and the previous layer. The specific difference equations are available in^65^ and the second derivatives can be expanded as

2 $$\begin{aligned} \phi ''_{ijk,l}= & {} 2 \left[ \frac{\phi _j}{(r_l-r_{l+1})(r_{l-1}-r_{l+1})} + \frac{\phi _i}{(r_{l+1}-r_l)(r_{l-1}-r_l)} + \frac{\phi _k}{(r_{l+1}-r_{l-1})(r_l-r_{l-1})} \right] \nonumber \\ z''_{ijk,l}= & {} 2 \left[ \frac{z_j}{(r_l-r_{l+1})(r_{l-1}-r_{l+1})} + \frac{z_i}{(r_{l+1}-r_l)(r_{l-1}-r_l)} + \frac{z_k}{(r_{l+1}-r_{l-1})(r_l-r_{l-1})} \right] \end{aligned}$$

where the role played by the link weights is apparent. The link weights encode the radial distances between consecutive layers. Apart from an overall normalization factor which is irrelevant, the link weights differ from unity only to the extent that the radial distances between the sensor layers are not equal.

In order to optimally combine the information from the azimuthal and longitudinal views, the difference in the respective resolutions must be taken into account by incorporating the appropriate relative normalization between the two terms in the $\Box _{ijk,l}$ operator. We have incorporated this relative normalization factor, which is reflected in the similarity of track quality metrics between the two views as discussed below.

This method differs in important ways from other investigations of track triggers^23–40^. One set of proposals being pursued primarily on the ATLAS experiment relies on pattern-matching using associative memories (AM). Our method is not based on pattern-matching and therefore does not require prior pattern generation and storage, which confers an immediate advantage because our method can be implemented on commercial, user-programmable integrated circuits (IC). In contrast, pattern-matching requires custom-designed AM ICs^23–28^. Such custom AM ICs have only been deployed once, in the CDF experiment at the Fermi National Accelerator Laboratory during the 1999–2011 data-taking period (see^68^ for a review). Plans to deploy a similar pattern-matching track trigger on the ATLAS experiment during the 2022–2024 data-taking period have been cancelled due to technical difficulties with the custom AM ICs and hardware. The second benefit of our method is its flexibility; if the number of sensor layers is changed, reprogramming the commercial ICs is straightforward, while the custom AM ICs need a major redesign. Thirdly, the pattern-matching method has only been pursued for the large-radius silicon-strip detector^23–29^. In contrast, our method can be deployed equally well on the small-radius (pixel) or large-radius (strip) sensors. We have demonstrated above that triggering on metastable particles in the highly-motivated range of sub-nanosecond lifetimes benefits enormously from a small-radius pixel-based trigger, which has never been investigated with the pattern-matching method. This gives our method a strong advantage in physics reach. The fourth and important physics benefit of our method is that it is intended to operate much faster than the pattern-matching method, such that it can trigger on a disappearing track with no requirement on any other detector. In other words, our method is amenable to a “first-level” track trigger with a latency less than $1\,\upmu$s^65^, which is compatible with the $4\,\upmu$s upper limit set by the HL-LHC experiments^69^. In contrast, the pattern-matching AM method has a latency of many tens of $\upmu$s^23^, making it incompatible with the first-level trigger. As such, it is being pursued as a second-level trigger following a calorimeter- or muon-based first-level trigger^23–29^, negating its efficacy as a disappearing-track trigger.

Variants of the above scheme have been considered^30,31^ but they suffer from the same disadvantages compared to our method. For example, the pattern-matching AM approach could be replaced with other pattern-recognition approaches such as the Hough transform^30^ or supervised learning methods such as deep learning^32–34^. The latter can be implemented in commercial ICs, eliminating the reliance on custom AM ICs and reducing the technical risk. However, the physics advantages of our method remain, because these variants are still based on the large-radius tracker, and still rely on tracker regions of interest defined by the particle traces in the calorimeter or muon detectors. Furthermore, they are intended to provide second-level triggering. For these reasons, they do not provide a standalone first-level trigger on short-lived metastable particles, and none of the corresponding physics gains that our method does.

Another key aspect of our method is that it does not require a customized detector geometry. An alternate method being pursued primarily on the CMS experiment^29,35–40^ relies strongly on track-trigger primitives supplied by the detector in the form of directional *stubs* built from pairs of hits. Computation will be performed on commercial ICs. Compared to the ATLAS methods mentioned above, this stub-based method is expected to execute faster and enable a first-level trigger. However, its tradeoff is that these stubs require sensor layers to be arranged radially as closely-spaced pairs. The negative implications of this detector geometry on other aspects of tracker performance have been deemed undesirable by the ATLAS Collaboration, who are not pursuing this option^69^. Our method has no such restriction and can be applied to any detector geometry, including strip and pixel sensors of arbitrary dimensions and radial placement. In particular, our method allows the detector geometry to be optimized on other experimental criteria, such as better momentum resolution and lower misidentification rates, and still provide a first-level track trigger. Our second physics advantage is again that this stub-based method is being pursued for the large-radius tracker only, since the small-radius tracker will not have the pairwise radial placement required by this approach.

These comparisons with other track-trigger investigations show that our approach is the first to promise a standalone first-level track trigger using the small-radius silicon sensors, that can access short-lived charged particles (down to 10 ps lifetime) without the rate limitation of an ISR trigger.

We note that a first-level hardware trigger needs access to all of the detector information from each event, i.e. the detector readout bandwidth needs to be compatible with the 40 MHz beam-crossing rate at the LHC. While this capability has not yet been deployed by ATLAS and CMS for the silicon tracking detectors, it has been demonstrated by the LHCb experiment’s upgrade of its silicon-pixel vertex detector^70^. One of the goals of this paper is to motivate the ATLAS and CMS experiments to consider a similar high-bandwidth readout for the HL-LHC. The motivation is provided by our silicon-based trigger that could process the data at this rate.

## Detector and event model

We demonstrate the algorithm by performing an emulation in software. We generate a point cloud from the intersections of 200 particles traversing 5 cylindrical sensor layers spaced 5 cm apart in a 2 T magnetic field, over an azimuthal domain of $2\pi$ and a longitudinal length of $\pm 1$ m. This detector geometry is representative of the LHC detectors ATLAS^5^ and CMS^6^, which employ a cylindrical spectrometer with a magnetic field of 2 T and 3.8 T respectively.

**Figure 6:**
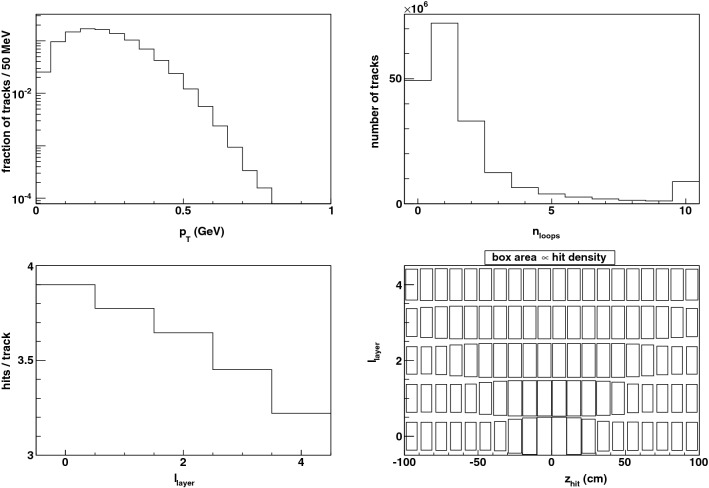
(top left) The generated distribution of the transverse momentum of pileup particles. (top right) The number of loops executed by the pileup particles in the magnetic field while traversing the barrel pixel layers. The last bin contains the overflow. (bottom left) The average number of hits deposited per layer by a pileup particle. (bottom right) The density of hits for each sensor layer as a function of the longitudinal coordinate.

The transverse momentum ($p_T$) spectrum in the emulation is realistically soft with a peak near $p_T \sim 250$ MeV^71^, as shown Fig. 6. The luminous region of the beam crossing is taken to be $\pm z_{{\rm luminous}}$ with $z_{{\rm luminous}} = 15$ cm and the $z_0$ values are uniformly distributed in this interval. We generate 6000 “pileup” particles per event, based on the production of 7 charged particles per collision per unit of pseudorapidity ($\eta$), 200 collisions per event, and a pseudorapidity coverage of $| \eta | < 2.1$ for the pixel barrel detector. The particles are distributed uniformly in azimuth and pseudorapidity, and we record all hits generated by them. 10% of all particles are assumed to be kaons and the rest to be pions. A fraction of these mesons, given by their time-dilated lifetimes, are decayed at a distance along their trajectory as determined from an exponential distribution. In these decays-in-flight, the daughter muon is propagated from its point of origin according to its momentum generated in the isotropic decay in the rest frame of the parent meson. Low-momentum particles perform multiple loops in the tracking detector, depositing hits in each helical loop. The number of loops executed by the particles is shown in Fig. 6. As a result, the number of hits deposited by a particle in the sensor layers is significantly more than unity, increasing from $\approx 3$ on the outermost layer to $\approx 4$ on the innermost layer.

We embed high-$p_T$ signal “trigger” particles of unit charge and with $p_T > 10$ GeV in this set of pileup hits. The signal $p_T$ spectrum is chosen to be uniform in $p_T^{-1}$ and the $\eta$ distribution is also uniform. For all particles (signal and pileup), the reduction of momentum due to ionization energy loss at each sensor layer is implemented, as is multiple Coulomb scattering which deflects the particle direction by an amount dependent on the momentum and the radiation lengths traversed. The radiation lengths of each sensor layer at normal incidence is taken as 4%^5^. The hits are smeared uniformly by up to $\pm 5\,\upmu$m in the azimuthal direction and $\pm 10\,\upmu$m in the longitudinal direction. Hits closer than twice the pixel $(r\phi ,z)$ dimension of $(25,50)\,\upmu$m^55,56^ are merged to emulate the passage of multiple particles through the same or adjacent pixels.

## Algorithm emulation

The resulting hit set is parsed into two-dimensional “towers”. First, conical rings are defined by the longitudinal boundaries $\left[ -z_{{\rm luminous}} - w_\lambda r, z_{{\rm luminous}} + w_\lambda r \right]$ at each sensor layer’s radius *r*, where $w_\lambda = 0.5$ is a tunable road width. Each conical ring is further sliced into azimuthal wedges such that each wedge contains *N* hits. In the realistic implementation of this parsing scheme, fast preprocessors will be used to stream the hit collection into these towers, with *N* as a tunable parameter to optimize the tradeoff between speed and the amount of processing circuitry. In this study we use $N = 16$ as a test case.

**Figure 7:**
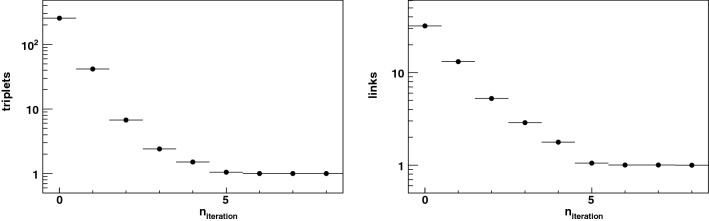
The exponential reduction in the number of hit triplets (left) and linked hit pairs (right) per node associated with a hit, at the end of each sort-cycle iteration (zero indicates the first iteration).

The hits within each tower are processed by an $N \times 3$ array of identical processing nodes which can be implemented in a field-programmable gate array (FPGA). Each node is associated with one hit $h_{i,l}$ in the middle layers (excluding the innermost and outermost layers) and is equipped to perform three functions; a difference engine, a sorting engine, and a scan engine. In our test case, an FPGA would contain 48 replicas of the circuits that implement these functions. A total of $\mathcal O(2000)$ FPGAs can process a full event. Since all $\mathcal O(10^5)$ processing nodes operate simultaneously, this massively parallel architecture can achieve low latency and high throughput.

The difference engine computes all first and second derivatives of the azimuthal and polar coordinates with respect to the layer radius. On the graph of hits, this corresponds to weighted differences where the weights are fixed by the detector geometry. Therefore the difference engine produces a list of $N \times N$ values of the $\Box _{ijk,l}$ operator, for the N values each of the *j* and *k* hit indices.

The criterion for the smoothest trajectory through any hit is the minimization of the two-dimensional $\Box$ operator. The sort engine sorts the $N \times N$ list of $\Box _{ijk,l}$ values in increasing magnitude. Each $\Box _{ijk,l}$ value is stored as part of a tuple containing the associated *j* and *k* values which identify the corresponding triplet of hits.

Next, the sorted list of tuples is used by the scan engine to create a ranked list of *j* and *k* values, where the rank is defined as the ordinal number of first appearance in the sorted $\Box _{ijk,l}$ list. Thus, a *j* or *k* value with a large rank is one that never makes a smooth trajectory, while a low rank corresponds to a smoother trajectory. In each sort cycle, the *j* and *k* values with large rank are dropped, which purges those links that are unlikely to form smooth trajectories. The number of links to be dropped at this step is a tunable parameter. For the results presented below, we drop half of the links that are present at the beginning of the sort cycle. In principle, a smaller drop fraction makes the algorithm more robust, while a larger drop fraction reduces the number of sort cycles required and increases the execution speed. We find that a link drop fraction of 50% provides a good tradeoff since the performance is robust and the total number of sort cycles is small.

**Figure 8:**
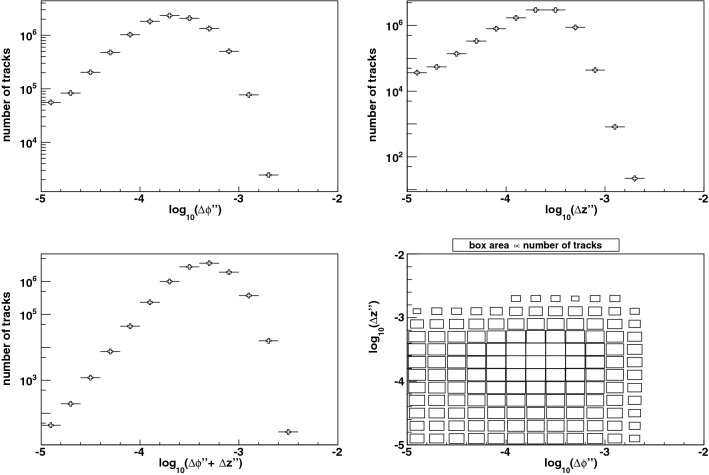
Distributions of reconstructed track quality metrics for signal tracks; (top left) smoothness metric $\Delta \phi ''$ in the azimuthal projection, (top right) smoothness metric $\Delta z''$ in the longitudinal projection, (bottom left) overall smoothness metric $\Delta \phi '' + \Delta z''$, and (bottom right) distribution of $\Delta z''$ versus $\Delta \phi ''$.

The three steps above constitute a sort cycle. Each node performs the sort cycle synchronously with all other nodes. In the next sort cycle, the reduced set of valid links at each node is used to remake the sorted $\Box _{ijk,l}$ list and the ranked link list, by re-engaging the sort and scan engines. Even though the link drop fraction is 50%, the length of the list is reduced by more than a factor of two, because nodes at subsequent and prior radii have also pruned links that do not correspond to smooth trajectories. The number of sort cycles required to purge bad links is logarithmic in the number of initial combinatorics, which makes this algorithm efficient. Furthermore, the sorting granularity may be increased geometrically with each sort cycle, allowing the design of the sort engine to be fast and efficient in resource usage. The exponential reduction in the number of viable (*k*, *i*, *j*) triplets at each node *i*, and the corresponding reduction in the number of viable links (*k*, *i*) and (*i*, *j*) between hit $h_{i,l}$ and hits $h_{k,l-1}$ and $h_{j,l+1}$, are illustrated in Fig. 7. About 7 sort cycles are needed to converge on the smoothest trajectory through each hit, consistent with $\log _2 (N^2)$ where $N=16$ is the number of hits in each layer processed by one FPGA.

The sequence of sort cycles terminates when the minimum $\Box _{ijk,l}$ values at all nodes are below a threshold which depends on the hit resolutions in the two dimensions. The end product of the sort-cycle stage is a linked tree of hits where each linked list from one sensor layer to the next is a potential trigger track. A hit may be shared by multiple linked lists, which is allowed at this stage since multiple particles may pass through the same pixels.

We define track quality metrics in each dimension by comparing the three values of $$\phi '' $$ evaluated at each of the three middle layers for a track candidate. The difference $$\Delta \phi ''$$ between the largest and smallest of these three (signed) values is a measure of the overall smoothness of the trajectory and its consistency with the azimuthal projection of a helix. Similarly, the difference $$\Delta z''$$ between the largest and smallest signed values of $$z'' $$ evaluated at the three middle layers is a measure of the smoothness and consistency with a helix in the longitudinal projection. Correctly-reconstructed signal particles have small values of $$\Delta \phi ''$$ and $$\Delta z''$$. Occasionally, additional track candidates exist with larger values of these quality metrics. Appropriate thresholds on these metrics eliminate the spurious candidates while maintaining high efficiency for the true high-$$p_T$$ particles. These thresholds depend on the pixel dimensions and hit resolutions, with a smaller dependence on the multiple Coulomb scattering.

**Figure 9:**
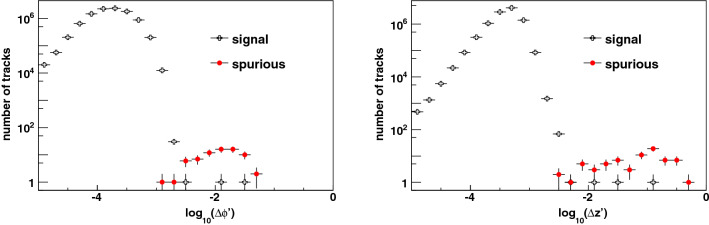
Distributions of consistency metrics $\Delta \phi '$ (left) and $\Delta z'$ (right) for tracks from a sample of 10 million signal particles, and spurious tracks from a sample of 20,000 simulated bunch crossings.

A second stage of pruning is performed by applying these thresholds on the track quality metrics, which removes spurious candidates and cuts off branches of the tree until high-quality tracks remain. The distributions of the quality metrics after this stage are shown in Fig. 8. The final consistency test of track properties is provided by the differences $\Delta \phi '$ and $\Delta z'$ between the largest and the smallest of the four signed values of $\phi '$ and $z'$ respectively. Correctly-reconstructed signal particles have small values of $\Delta \phi '$ and $\Delta z'$, while spurious track candidates tend to have large values of these consistency metrics. We study the properties of spurious tracks from our emulation without embedding the high-$p_T$ particle amongst the pileup hits, thus simulating bunch crossings of the colliding beams which produce 200 pileup collisions only. The distributions of these consistency metrics (Fig. 9) show that the requirements $\Delta \phi ' < 0.005$ and $\Delta z' < 0.005$ suppress the spurious track rate substantially, with negligible loss of signal efficiency.

Since the calculation of derivatives has already been performed, a well-defined curvature is obtained for a 1-to-1 linked list by averaging the four values of the azimuthal first derivatives. A trigger decision on a high-$p_T$ track can be made at this stage by defining a threshold on this average curvature value, which is equivalent to $p_T^{-1}$.

The algorithms presented here and in Ref.^65^ are both based on concepts of graph computing, but are substantially different in detail. The method of Ref.^65^ was simplistic and unable to process sensor data unless the following conditions were strictly satisfied: (i) each truth-level particle deposited a hit in each layer, (ii) proximate hits from different truth-level particles were never merged (iii) every hit was deposited by a truth-level particle (ie. there were no noise hits), (iv) all truth-level particles were within the acceptance of the slice of the detector being processed, and (v) particles did not decay in flight or execute multiple loops in the magnetic field. These limitations restricted that algorithm to the idealized situations depicted in Fig. 1 of Ref.^65^.

The algorithm presented in this paper has been re-invented to be able to process an arbitrary collection of hits. There is no restriction on the origin of the hits, which can include noise, decaying particles, photon conversions and secondary interactions, and particles executing an arbitrary number of loops in the magnetic field. The hit collection may or may not contain a reconstructable trajectory from a truth-level particle; the new algorithm is unbiased and agnostic from this perspective, and does not suffer from any of the above limitations.

**Figure 10:**
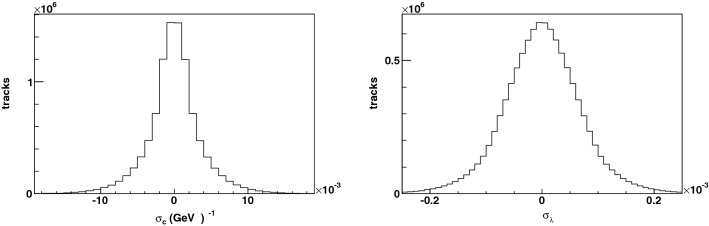
Distribution of (left) the difference of $q/p_T$ between the reconstructed and true particle, yielding a curvature resolution $\sigma _c = 3.7$ TeV$^{-1}$, and (right) the difference of $\lambda$ between the reconstructed and true particle, yielding a resolution on the cotangent of the polar angle $\sigma _\lambda = 7 \times 10^{-5}$.

Another limiting aspect of the algorithm in Ref.^65^ was that it could only process one-dimensional hits, though its extension to two dimensions was mentioned as potentially realizable. The algorithm presented here is flexible and can be configured for one-dimensional (ie. silicon strip) or two-dimensional (ie. silicon pixel) hits. In two dimensions, the new algorithm can accommodate different resolutions in the longitudinal and transverse views. Thus, the algorithm presented here is deployable under realistic conditions. It also supports the notion of spurious tracks in an unbiased fashion, unlike Ref.^65^ where the inadmissibility of spurious hits resulted in a narrow interpretation of spurious tracks.

## Results

The track parameter resolutions are shown in Fig. 10. The resolution on $q/p_T$ is $\approx 4$ TeV$^{-1}$ versus $\approx 0.4$ TeV$^{-1}$ for the ATLAS tracker. The ratio is close to the expected ratio of $\approx 12$ based on the inverse proportionality to the square of the track length (25 cm in this study and 1 m for the ATLAS tracker) and to the square root of the number of hits (5 in this study and $\sim 12$ for the ATLAS tracker), and based on the proportionality to the position resolution (25 $\upmu$m in this study and 50 $\upmu$m in ATLAS).

**Figure 11:**
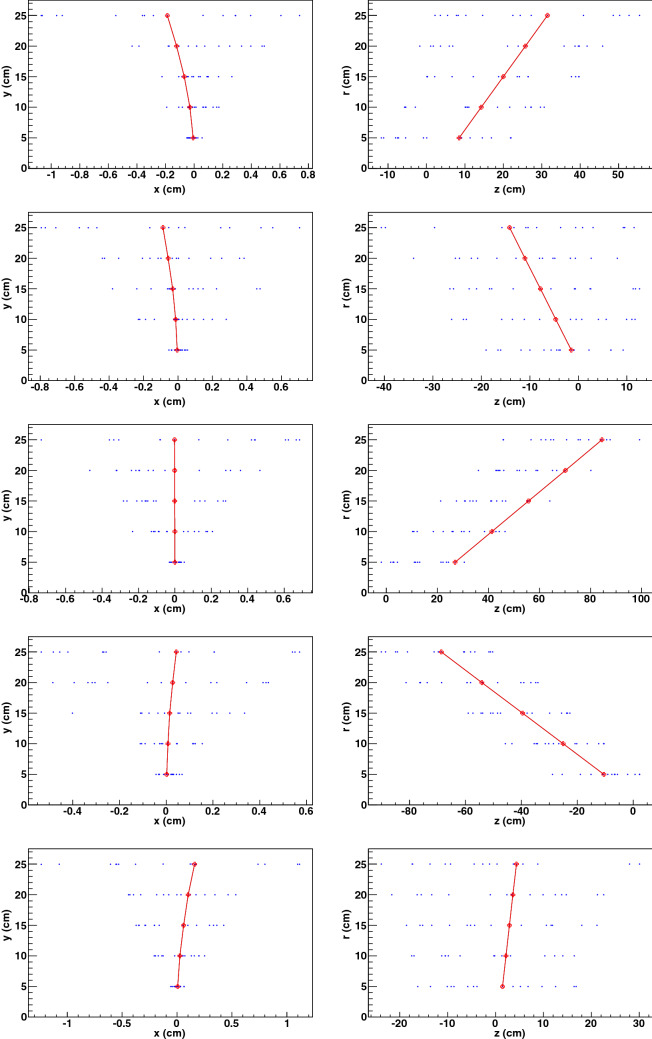
Five examples of events showing the high-$p_T$ signal track (red curve) found amongst the pileup hits (blue dots) in the transverse (left) and longitudinal (right) views. The red points denote hits created by the signal particle.

**Figure 12:**
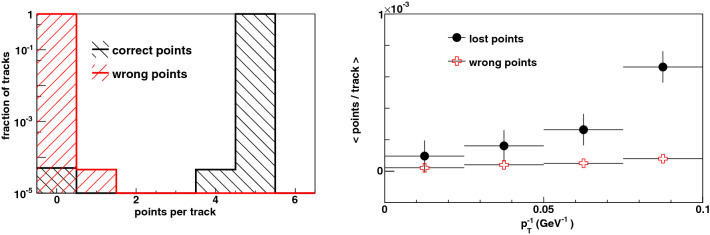
The number of correctly and wrongly assigned hits per track for signal particles with $p_T > 10$ GeV, shown (left) inclusively and (right) as a function of true $p_T^{-1}$.

Examples of signal tracks found amongst the pileup hits are shown in Fig. 11. Each hit on a reconstructed trajectory is compared to its progenitor particle trajectory, and the number of correctly assigned hits and wrongly assigned hits per trajectory is shown in Fig. 12. We note that a very large fraction of the tracks have the maximum of 5 hits correctly assigned, and very few tracks have wrongly assigned hits. The rate of loss of correct hits (ie. the inefficiency) and the rate of assignment of spurious hits both slowly increase with curvature, also shown in Fig. 12. The inclusive efficiency for finding particles with $p_T > 10$ GeV is $(99.995 \pm 0.001_{{\rm stat}})$%.

For a viable trigger it is important that the rate of spurious high-$p_T$ tracks be sufficiently low, lest it overwhelm the bandwidth of the trigger and data acquisition (TDAQ) system. Typically, the production rate for the events of interest is much lower than the rate of spurious triggers and the latter dominate the TDAQ bandwidth. In 20,000 simulated bunch crossings, and without applying the consistency metric thresholds, 28 tracks with $p_T > 5$ GeV are found whose reconstructed $p_T$ spectrum is uniform in $p_T^{-1}$ within statistics. Thus we estimate a spurious trigger rate per $p_T^{-1}$ interval per bunch crossing of $(7 \pm 1_{{\rm stat}}) \times 10^{-3} \frac{p_T}{\mathrm{GeV}}$, which corresponds to an average of one spurious trigger with $p_T > 10$ GeV in 1,400 bunch crossings. For a 25 ns bunch crossing time (40 MHz), the spurious trigger rate is thus estimated to be 28 kHz, which is already much smaller that the budgeted first-level hardware trigger rate of 1 MHz at ATLAS^57^ and 750 kHz at CMS^58^ at the HL-LHC. The spurious trigger rate depends on the thresholds placed on the track quality metrics $\Delta \phi ''$ and $\Delta z''$, the consistency metrics $\Delta \phi '$ and $\Delta z'$, and on the pixel dimensions and sensor alignment. No spurious tracks satisfy the consistency metric requirements $\Delta \phi ' < 0.005$ and $\Delta z' < 0.005$, which reduce the spurious trigger rate dramatically to $\mathcal O$(1 kHz), equivalent to a spurious trigger in $\mathcal O$(40,000) bunch crossings. Studies are in progress to characterize the spurious rate in more detail. These and other metrics could be compared to other approaches such as geometric deep learning^67^ applied to tracking^34^.

In conclusion, we have developed a methodology to rapidly identify high-$p_T$ charged particles produced in proton-proton collisions at the HL-LHC and future colliders. By using information from silicon pixel sensors placed at small radius from the colliding beams, our method can be applied to trigger on meta-stable charged particles which decay invisibly before traversing other detectors, thereby accessing a motivated region of parameter space in the particles’ lifetime and mass. The trigger efficiency is essentially 100% for $p_T > 10$ GeV and the $p_T$-resolution of 4% at $p_T = 10$ GeV implies a sharp trigger turn-on. The method, based on graph computing techniques, can be considered a form of unsupervised machine learning since it performs clustering of data points according to expected patterns without training data. It is designed to operate in the noisy environment in which the patterns of interest are embedded, and with one- or two-dimensional sensor geometries of various resolutions. Simulations under realistic conditions show that the method achieves a high signal efficiency and a low spurious rate which is well within the budgeted bandwidth. In future studies we plan to investigate alternate detector geometries and the impact of systematic deformations on the algorithm performance, as well as the processing speed and resource requirements of an FPGA implementation.

## Data Availability

The datasets generated during and/or analysed during the current study are available from the author on reasonable request.

## Code availability

The custom computer code written for this algorithm is available from the author on reasonable request.
